# Starvation Induces Phenotypic Diversification and Convergent Evolution in *Vibrio vulnificus*

**DOI:** 10.1371/journal.pone.0088658

**Published:** 2014-02-13

**Authors:** Hwajiun Chen, Chun-Yao Chen

**Affiliations:** 1 Department of Life Science, Tzu-Chi University, Hualien, Taiwan; 2 Institute of Medical Sciences, Tzu-Chi University, Hualien, Taiwan; State Key Laboratory of Pathogen and Biosecurity, Beijing Institute of Microbiology and Epidemiology, China

## Abstract

Starvation is a common stress experienced by bacteria living in natural environments and the ability to adapt to and survive intense stress is of paramount importance for any bacterial population. A series of starvation experiments were conducted using *V. vulnificus* 93U204 in phosphate-buffered saline and seawater. The starved population entered the death phase during the first week and approximately 1% of cells survived. After that the population entered a long-term stationary phase, and could survive for years. Starvation-induced diversification (SID) of phenotypes was observed in starved populations and phenotypic variants (PVs) appeared in less than 8 days. The cell density, rather than the population size, had a major effect on the extent of SID. SID was also observed in strain YJ016, where it evolved at a faster pace. PVs appeared to emerge in a fixed order: PV with reduced motility, PV with reduced proteolytic activity, and PV with reduced hemolytic activity. All of the tested PVs had growth advantages in the stationary phase phenotypes and increased fitness compared with 93U204 cells in co-culture competition experiments, which indicates that they had adapted to starvation. We also found that SID occurred in natural seawater with a salinity of 1%–3%, so this mechanism may facilitate bacterial adaptation in natural environments.

## Introduction

*Vibrio vulnificus* is a notorious human pathogen, which causes clinical manifestations that range from wound infection to primary sepsis, or even death [1]. This species is also a fish pathogen that affects eel and tilapia aquaculture in many Asian and European countries, including Japan [2], Bangladesh [3], Denmark [4], and Spain [5]. It has also been isolated from oysters and shrimps [6], [7].

However, *V. vulnificus* is usually found in aquatic environments as free-living bacteria. Environmental *V. vulnificus* isolates are reported to be highly heterogeneous. Arias et al. reported high genetic diversity after ribotyping 132 strains of *V. vulnificus* by random amplified polymorphic DNA PCR [8]. Wong et al. also demonstrated high genetic heterogeneity in environmental isolates from the United States and Taiwan using pulsed-field gel electrophoresis [9]. The mechanism that allows this high genetic diversity to be maintained in marine environments still remains unknown. This high genetic heterogeneity may be derived from a continuous and abundant supply of mutations in natural populations, or it could be the result of relaxed selection in a heterogeneous environment.

In natural environments, bacteria are expected to experience repeated “feast or famine” fluctuations, so the capacities to survive starvation and respond rapidly to transient nutrient bursts are of paramount importance. Gram-negative bacteria react to various types of environmental stress with a general stress response. This response depends on alternative sigma factors such as RpoS, which regulate a suite of genes and redirect resources from supporting growth to self-maintenance (reviewed in [10]). These include genes related to metabolic adaptations to available resources and alterations in the control of motility, cell morphology, stress resistance, and biofilm formation.

Bacteria activate the general stress response early during starvation. They will induce further stringent responses if the nutritional limitation persists and the cells will stop to grow, while there is a switch to a more error-prone DNA polymerase, which may elevate the mutation rate [11]. Stress-directed mutation is considered a universal mechanism of microbial adaptation to environmental stress at the population level during long-term starvation [12]. This mechanism may contribute to adaptive evolution in *Escherichia coli* in laboratory culture [13]. This phenomenon has been described in many microbes in laboratory settings, but its occurrence in natural habitats has not been examined previously.

The population dynamics of *V. vulnificus* in conditions of starvation have been examined in several studies. Biotype 2 strain E22 *V. vulnificus* was reported to survive starvation for 50 days in artificial seawater [14]. In a subsequent study, two- to three-order drops in population size were seen in E22 after 160-day starvation in water with 0.5% or 1.5% salinity at 25°C [15]. Similarly, approximately 1% of biotype 1 strain C7184 cells survived at the end of 35-day starvation in artificial seawater at 22°C [16]. The question is how does starvation affect the physiology of *Vibrio* spp. and does it affect bacterial diversification? Many *Vibrio* spp. can survive long-term starvation, even for several years [15], [17]. They enter a viable but non-culturable (VBNC) state when they are starved at a low temperature [18]. The starved cells are known to change their morphology from rod to coccoid cells, as reported in *V. vulnificus* [15], *V. parahaemolyticus* [19], *V. angustum* [20], *V. shilloi,* and *V. tasmaniensis* [21]. Starved *V. parahaemolyticus* cells also exhibit greater cell adherence and hydrophobicity [9]. These results indicate that starvation can lead to dramatic changes in the morphology and physiology of *Vibrio* spp.

Previously, we isolated *V. vulnificus* from diseased tilapia [22]. The epidemics occurred only in fish maintained in a low salinity (<0.5%) environment, which suggests that these tilapia-pathogenic isolates were descendants of low salinity-adapted *V. vulnificus*. Further analysis indicated that these isolates formed several genetic groups (unpublished data), which indicates either that the ability to infect tilapia has evolved more than once, or that horizontal gene transfer is responsible for the emergence of novel pathogenic strains. We hypothesize that if starvation can lead to phenotypic diversification in a natural environment, it may facilitate the generation of virulent bacterial variants by providing a larger pool of diverse progeny for selection. The aim of the present study was to show that starvation can induce the diversification of *V. vulnificus* in laboratory and natural settings, and that this starvation-induced diversification (SID) could promote bacterial adaptation and persistence. We showed that *V. vulnificus* survived for an extended period and exhibited phenotypic diversification. SID was cell density-dependent and a result of adaptive mutation. We provide evidence to demonstrate that SID occurred with various salinity levels and was observed in two different isolates.

## Materials and Methods

### Bacterial strains, culture media, and growth conditions

All of the bacterial culture experiments were performed at 30°C. Bacteria were grown in tryptic soy broth or agar supplemented with 1.5% NaCl (TSBS and TSAS, respectively). Strain 93U204 is a fish pathogen, which was isolated from moribund tilapia collected from Kaohsiung in Taiwan. This isolate was a kind gift from Dr. Chia-Ben Chao of Kaohsiung Institute of Livestock Disease Control and Prevention, and was identified as *V. vulnificus* by 16S rDNA sequencing (manuscript in preparation). YJ016 was a human clinical strain isolated from Tainan, Taiwan, and its genome has been sequenced [23]. This strain was kind gift from Dr Lien-I Hor of Department of Microbiology and Immunology, National Cheng-Kung University, Taiwan. All bacterial isolates were properly stored by putting 100 µL overnight culture in 1 mL of TSBS supplemented with 50% glycerol, and then kept in –80°C freezer (GV039P/M, Kaltis International, Taipei, Taiwan).

### Long-term starvation of bacterial cultures

Bacteria were grown in TSBS with constant 200 rpm horizontal shaking (E450, Deng Yng, New Taipei City, Taiwan). Cells were harvested in the late exponential phase (approximately 1×10^9^ CFU/mL), washed twice with phosphate-buffered saline (PBS, 0.1 M phosphate buffer and 0.85% NaCl, pH 7.3), and re-suspended in suitable media. The bacterial cells were diluted in PBS to the desired concentrations if needed.

Most of the starvation experiments were conducted in 30-mL Wheaton glass serum bottles (Z113980, Wheaton, Millville, New Jersey, USA). Experiments conducted in other containers are indicated. The cells were washed twice with PBS, and re-suspended in 10 mL test media. Each serum bottle was capped with a flange red rubber stopper (224100-172, Wheaton) and sealed with an open aluminum cap (224178-01, Wheaton). The cells were incubated at 30°C without shaking and were sampled repeatedly using 1-mL insulin syringes (27-gauge, Terumo, Tokyo, Japan). The viable counts were determined using the drop plate method [24]. The bacterial suspensions were serially diluted in TSBS and five 10 µL drops of each dilution were placed on TSAS plates. The plates were incubated at 30°C for 18 h before they were counted. We chose to use TSBS as diluent to prevent possible loss of viability of these stressed cells on agar plate, which has been documented for *V. vulnificus* [25]. The serial dilution process generally took less than 10 minutes to perform, and in a preliminary test, cells starved in PBS did not increase significantly (n = 6, paired t test, P>0.05) after being transferred into TSBS for 20 minutes. Therefore we consider using TSBS as diluent did not invalidate the drop plate results. To compare the bacterial survival in various media, 93U204 cells were re-suspended in PBS, natural seawater with 3% salinity (NSW), half-strength natural seawater diluted with distilled water (HNSW), or TSBS. The salinity of seawater was determined using a hand-held salinity refractometer (MR100ATC, Milwaukee Instruments, Rocky Mount, North Carolina, USA). The starting concentration of 93U204 was approximately 5×10^9^ CFU/mL. The survival rate was determined every day for 7 days by plate counts on TSAS after serial dilution. In the long-term starvation experiment, we re-suspended the cells in PBS or TSBS for 214 days using a starting concentration of 1×10^9^ CFU/mL. The bacterial viability was measured on days 0, 1, 2, 3, 8, 14, 35, 56, 93, 115, and 214.

### Phenotypic characterization

We developed a Motility-Hemolysis-Proteolysis (MHP) grouping system to classify *V. vulnificus* isolates, which was based on three readily identifiable characteristics, namely motility (M), hemolysis (H), and proteolysis (P). The isolates were classified into one of 100 phenotype groups according to their motility (five levels, from M0 to M4), hemolysis (four levels, from H0 to H3), and proteolysis (five levels, from P0 to P4) activity levels. Motility, hemolytic activity and proteolytic activity were measured on motility agar (1% tryptone, 2% NaCl, 0.25% agar), blood agar (LB agar supplemented with 5% sheep blood), and skim milk agar (LB agar supplemented with 2% skim milk). In every test the progenitor 93U204 was inoculated on the same test plate as a control, and the diameters of its colony on motility agar, clear zone on blood agar and clear zone on skim milk agar were recorded. We defined M0 to M4 as having colony size of 0, 0–0.33, 0.33–0.67, 0.67–1.2, or over 1.2 times of that measured in 93U204 control; H0 to H3 as having clear zone on blood agar of 0, 0–0.5, 0.5–1.5, or over 1.5 times of that measured in 93U204 control; and P0 to P4 as having clear zone on skim milk agar of 0, 0–0.33, 0.33–0.67, 0.67–1.2, or over 1.2 times of that measured in 93U204 control. The progenitor 93U204 had the M3H2P3 phenotype, and the human pathogenic strain YJ016 had the M3H1P3 phenotype. Some 93U204 phenotypic variants (PVs) developed opaque or translucent colonies. These PVs included an additional “O” for opaque and “T” for transparent in their MHP label.

To determine the phenotypic composition, colonies from each sample were picked randomly with sterile toothpicks and inoculated into 150 µL LB in a 96-well microtiter plate, followed by overnight incubation at 30°C. We used a stainless steel 96-pin replicator to transfer the bacterial cells onto motility agar, skim milk agar, blood agar, and thiosulfate-citrate-bile salts-sucrose (TCBS) agar. The results for motility, proteolytic activity, hemolytic activity, and growth on TCBS were determined at 6, 20, 48, and 20 h after inoculation at 30°C. Isolates with the same MHP grouping were defined as a PV.

### Starvation-induced diversification (SID) experiments

In this study six different SID experiments in PBS were conducted. Each bottle of inoculated PBS was treated as a population. Populations within the same experimental group received the same 93U204 preparation as the inocula. For each population, a sampling series was conducted at the indicated time for each experiment to trace the dynamics of phenotypic change. For each sample, approximately 30 colonies were isolated for phenotypic characterization and the result was used to calculate the relative abundance of each PV. The starting bacterial density was adjusted to approximately 10^9 ^CFU/mL, and the experiments were conducted in 10 mL of PBS in 30-mL serum bottles, unless indicated otherwise.

The settings for each experiment were as follows. (1) The analysis of temporal change in the PV composition included two sets of experiments, with one population in each set. Both populations received the same inoculum. Samples were taken on days 0, 8, 16, 24, and 32. (2) The analysis of SID in replicate populations comprised two sets of experiments, with five populations in each set. Samples were taken on day 33. (3) The SID and population survival rate analysis comprised 12 sets of experiments, with 1–3 populations in each set and a total of 20. Samples were taken on days 14 or 15. (4) The analysis of the effect of cell density on SID comprised four sets of experiments, with three populations in each set. The bacterial densities in these four sets were adjusted to approximately 10^9^, 10^7^, 10^5^, and 10^3^ CFU/mL. Samples were taken on days 14, 28, and 42. (5) The analysis of the effect of population size on SID comprised three sets of experiments, with three populations in each set. The bacterial suspension volumes used in the three sets were 100 mL, 10 mL, and 3 mL, in 125-mL serum bottles (Z114014, Wheaton), 30-mL serum bottles, and 30-mL serum bottles, respectively. Samples were taken on days 14, 28 and 42. (6) Two *V. vulnificus* strains, 93U204 and YJ016, were used to test the effect of salinity on SID. For each strain, the analysis comprised one set of experiments, with three treatments in each set. Samples were taken on days 14, 28, and 42. These sets used natural seawater with 1%, 2%, and 3% salinities. The seawater was sterilized using 0.22 µm filter. The salinity was checked using a refractometer. The desired salinity was achieved by mixing freshly collected seawater with freshwater from the same estuary area.

### Competition experiments

Three isolates, each from a separate PBS-starvation survivor population of 93U204, were used in the competition experiments. B33-12 was isolated from the trial B 33-day population; C30-2 was isolated from the trial C 30-day population; and D33-4 was isolated from the trial D 33-day population. All of the strains derived from the starvation cultures were identified as *V. vulnificus* by species-specific *vvp* PCR [26]. Another primer pair of p134/p135 was also used, which targeted *V. vulnificus rpoS* [27]. The *rpoS* primer pair comprised the forward primer p134 (5′-ACATAACgATAATTACCTCAgTgC-3′), which started 264 bp upstream of the YJ016 *rpoS*, and the reverse primer p135 (5′-CgTCAAAAATTgTCTCAACTTg-3′), which ended 276 bp downstream of YJ016 *rpoS* and was located in *mutS*. The expected size of this PCR product was 1530 bp. The PCR protocol comprised a preheating step at 94°C for 6 min, followed by 30 cycles of 94°C for 30 s, 55°C for 30 s, and 72°C for 2 min, with a final extension at 72°C for 7 min. Their *rpoS* DNA sequences were identical to those of the 93U204 progenitor cell according to nucleotide sequencing with an Applied Biosystems 3730xl DNA Analyzer, which was conducted at the VYM Genome Research Center of National Yang-Ming University. The obtained nucleotide sequences were compared with the GenBank DNA database using the BLASTn program.

All of the isolates were stored at −80°C in TSBS with 15% glycerol. The stock was subcultured in 12 mL TSBS in a 125-mL flask and grown to the late exponential phase with 200 rpm horizontal shaking. The culture was then washed twice with PBS by centrifugation (10 min, room temperature, 4500×*g*). The bacterial preparation was re-suspended in PBS to the desired concentration. The progenitor 93U204 was mixed at 1000:1 with each of the isogenic cultures derived from C30-2, D33-4, or B33-12 in PBS at the start of a two-week experiment. Monocultures of the 93U204 progenitor and mutants were included as controls.

The bacterial concentration of each sample was determined using the drop plate method with TSAS plates. The relative abundance of each competing population was determined every 2 days for 14 days. We used phenotypic characterization to identify the relative amount of the two competing phenotypes, because only a small fraction of the cells changed their phenotype up to the late stage of the starvation experiment. In each of these competition experiments, cells with reduced motility and enhanced proteolytic activity were considered to be descendents of C30-2, cells with reduced motility that were unable to grow on TCBS were considered to be descendents of D33-4, and cells with reduced motility and enhanced proteolytic activities were considered to be descendents of B33-12.

### Diversity evaluation and statistical analysis

The bacterial diversity was evaluated using the Shannon diversity index, *H*′  =  −∑*p_i_*ln*p_i_*, where *p_i_* is the relative abundance of each phenotype. Each phenotype was defined as having the same motility, hemolysis, and proteolysis activity characteristics. Cluster analysis and linear regression were performed using PAST software (http://folk.uio.no/ohammer/past/) [28].

### Electron microscopy

All of the electron microscopy experiments were conducted in the Electron Microscopy Center, Tzu-Chi University. A bacterial suspension from each preparation was centrifuged at 5,000×*g* for 5 min at 4°C to collect bacterial cells. The pellets were re-suspended and fixed with 2.5% glutaldehyde in PBS at 4°C. On the day the observations were made, the bacterial cells were washed twice with 0.1 M phosphate buffer with 5% sucrose, post-fixed with 1% osmium tetraoxide for 1 h, and washed twice with 0.1 M phosphate buffer with 5% sucrose. Next, a 5 µL drop of bacterial suspension was placed on a Formvar/carbon-coated grid for 2 min to allow absorption of bacteria onto the grid. A drop of 2% phosphotungstic acid was placed on the grid to achieve negative staining. The grids were then air-dried and examined using a Hitachi H-7500 transmission electron microscope (Hitachi, Tokyo, Japan) at 80 kV. Digital images were collected using a 2048×2048 Macrofire monochrome CCD camera (Optronics, Goleta, California, USA). Cell diameter and length measurements were made using these images with ImageJ v. 1.46 (http://rsbweb.nih.gov/ij/index.html).

### Chemicals and reagents

All of the PCR-related reagents were included in the Taq DNA Polymerase 2X Master Mix Red kit (1.5 mM MgCl_2_, Ampliqon IIII, Odense, Denmark). The electron microscopy chemicals and reagents were obtained from Electron Microscopy Services (Hatfield, Pennsylvania, USA). The culture media and ingredients were obtained from Becton Dickinson and Company (Sparks, Maryland, USA), while all other chemicals were obtained from Sigma (St Louis, Missouri, USA). All of the primers were purchased from Gene Messenger (Kaohsiung City, Taiwan). Fresh sheep blood was obtained from Creative Media Products Ltd (New Taipei City, Taiwan).

## Results

### Survival of *V. vulnificus* 93U204 after starvation in various culture media

The repeated appearance of new *V. vulnificus* pathogens in tilapia farms and their high heterogeneity in natural populations (unpublished data) suggests that some environmental biotype 1 strains might repeatedly invade and persist in low-salinity tilapia farms, which later became pathogens. Thus, we aimed to determine whether environmental stresses, such as starvation, could contribute to the emergence of virulence.

**Figure 1:**
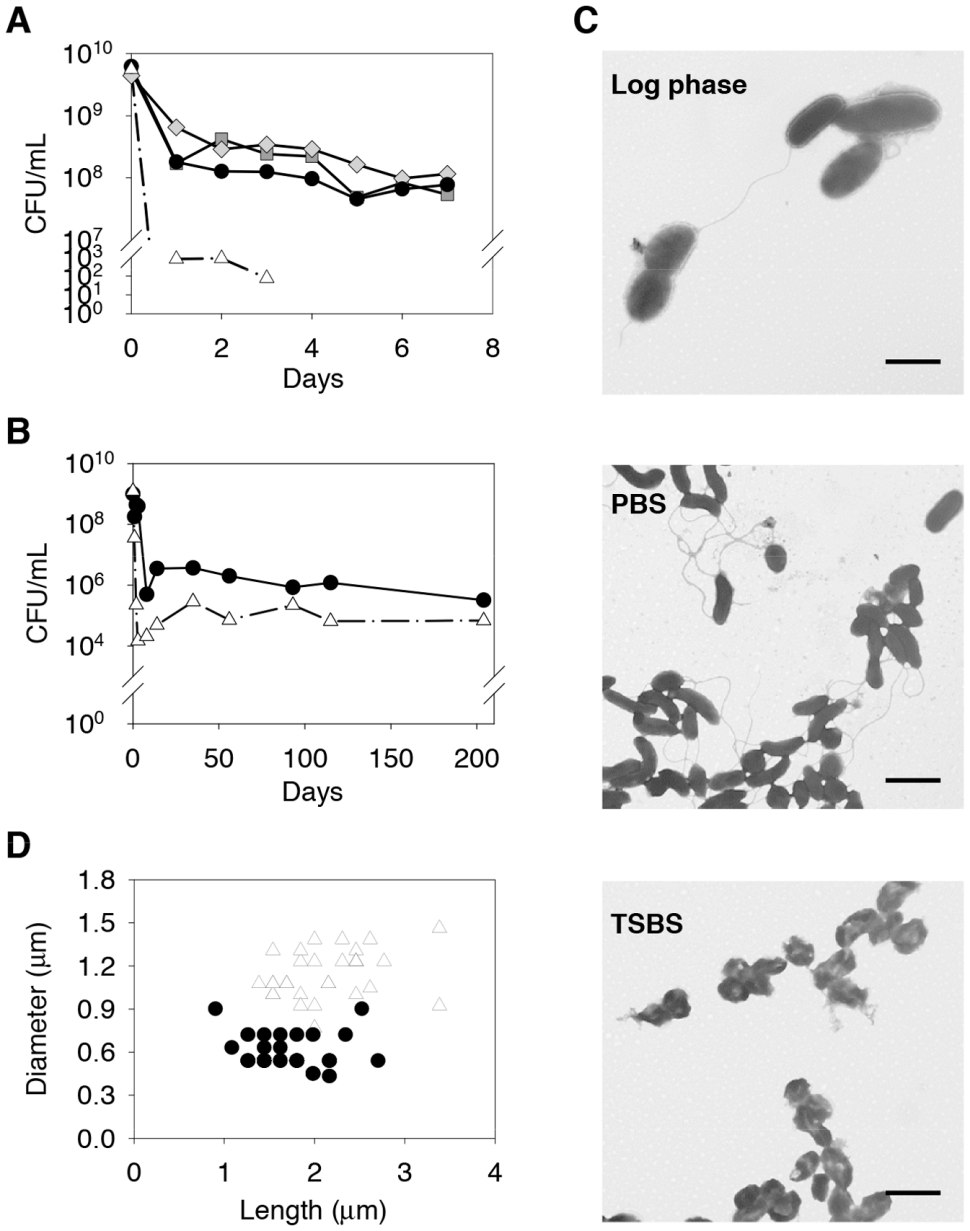
Survival of *V. vulnificus* cultures in various media. (A) Dynamics of *V. vulnificus* populations in phosphate-buffered saline (PBS, •), natural seawater (NSW, ◊), half-strength natural seawater (HNSW, □), and tryptic soy broth with 1.5% NaCl (TSBS, △) during the first week after inoculation. The results shown were derived from three replicate populations. (B) Dynamics of *V. vulnificus* populations in PBS (•) and TSBS (△) during an extended period of starvation (214 days). Representative results are shown from one experiment. (C) Electron micrographs of *V. vulnificus* cells showing the cellular morphology of a log-phase population (Log phase), a population starved in PBS for 4 days (PBS), and a population starved in TSBS for 4 days (TSBS). Most of the cells starved in TSBS were ruptured. All of the bars represent 2 µm. (D) The diameters and lengths of log-phase cells (△) and cells starved in PBS for 4 days (•).

First, we examined the survival of *V. vulnificus* 93U204 in nutrient-poor PBS and seawater, or nutrient-rich TSBS. The number of culturable 93U204 in PBS dropped rapidly by two orders from the starting concentration of 5×10^9^ CFU/mL during the first week in PBS, NSW and HNSW (Figure 1A). The viable population was even smaller in TSBS, to approximately 8×10^2^ CFU/mL on day 1 and below the detection limit (20 CFU/mL) after day 3.

To understand the long-term survival of *V. vulnificus*, we inoculated PBS and TSBS with cells at approximately 1×10^9^ CFU/mL, and tracked the viable population dynamics for 7 months (Figure 1B). Three-order and five-order drops were seen after 7 days in the PBS and TSBS groups, respectively. The mortality stabilized after 2 weeks, then the populations entered a slow and gradual declining phase in both groups. In another experiment with a starting cell density of 5×10^8^ CFU/mL, approximately 10^3^ CFU/mL of *V. vulnificus* survived starvation in PBS at 1215 days after inoculation. We conclude that a starved 93U204 population will typically experience a rapid decrease during the first week, before the mortality stabilizes after the second week. The population then enters a slow period of decline, but can survive for months to years.

We examined the morphology of starved cells by transmission electron microscopy (Figure 1C). The un-stressed log phase cells exhibited the typical vibrioid morphology, whereas the cells starved in PBS for 4 days became more slender. Most of the cells from the 4 day TSBS population were ruptured, which indicated their extremely low viability in this treatment. PBS-starved cells were a similar length to the log-phase cells, but their cell diameters were much smaller (Figure 1D).

### Starvation and PBS-induced phenotypic diversification in *V. vulnificus*

**Figure 2:**
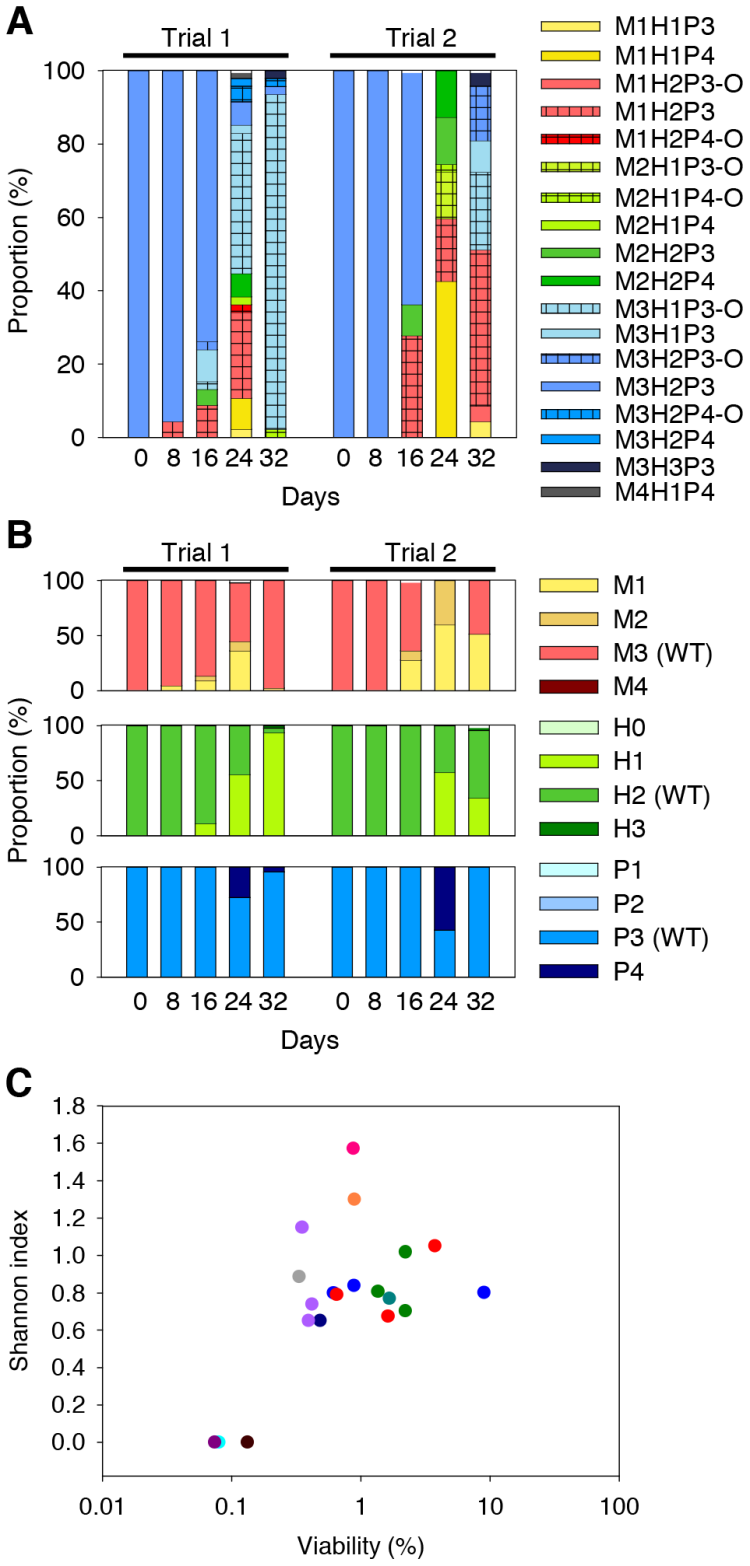
Phenotypic diversification of the *V. vulnificus* 93U204 population during starvation in phosphate-buffered saline. (A) Relative abundance of phenotypic variants in starved populations from two replicate trials. The population gradually diversified from the 93U204 M3H2P3 phenotype. (B) Compositions of populations, which were categorized based on their motility (top panel), hemolytic activity (middle), and proteolytic activity (bottom panel). WT, phenotype of the parental 93U204. (C) Association of diversity with mortality in starved populations. Twenty populations were starved in phosphate-buffered saline, and their survival and diversity were measured on days 14 or 15. The figure shows the distinction between low survival/un-diversified populations (left) and high survival/diversified populations. These 20 populations were derived from 12 separate inoculations (indicated by different colors).

The high mortality in starved *V. vulnificus* populations may exert strong selection on survivors. We conducted 2 independent PBS starvation trials of 93U204 and monitored the population for phenotypic change. SID was seen in both trials (Figure 2A). A total of 16 and 13 PVs were found in trial 1 and trial 2, respectively. We designated the progenitor 93U204 M3H2P3 phenotype as the “wild-type (WT)” phenotype. PV was first detected at day 8 in trial 1, and at day 16 in trial 2. The proportion of WT cells decreased with time as the number of detected PVs increased. To confirm that these changes were not transient, we picked five morphologically distinct colonies from each population to assess their phenotypic consistency through subcultures. After at least three subcultures onto TSAS, none reverted back to the WT phenotype, which demonstrated that these changes were not transient and were likely to be genetic.

The PVs in each population were categorized further to detect general trends in the motility, hemolysis, or proteolysis activity (Figure 2B). PVs with reduced motility (mot-R PVs) and with reduced hemolytic activity (hem-R PVs) increased with time, and the mot-R PVs appeared earlier than the hem-R PVs during starvation. PVs with enhanced proteolysis activity (pro-E PVs) appeared transiently on day 24 and disappeared or decreased greatly on day 32 in both trials. We conclude that a reduction in motility, a reduction in hemolytic activity, and an enhancement in proteolytic activity could promote the survival of 93U204 during starvation.

### SID was associated with cell survival in populations

If diversification is a bacterial strategy for overcoming environmental stress, there should be a correlation between diversity and population survival. Thus, we collected survival and PV composition data from 20 starved 93U204 populations. All of these populations were started with approximately 10^9^ CFU/mL in 10 mL PBS, followed by starvation for 14 or 15 days before sampling. Three of the 20 populations had very low viability at approximately 0.1% of the original population (Figure 2C). SID was seen in all but these three populations, which remained homogeneous and all of the colonies examined (30 for each population) had an unchanged phenotype. This result suggests that SID may be needed for the population to overcome environmental stress.

### Source of PVs in starvation survival populations

**Figure 3:**
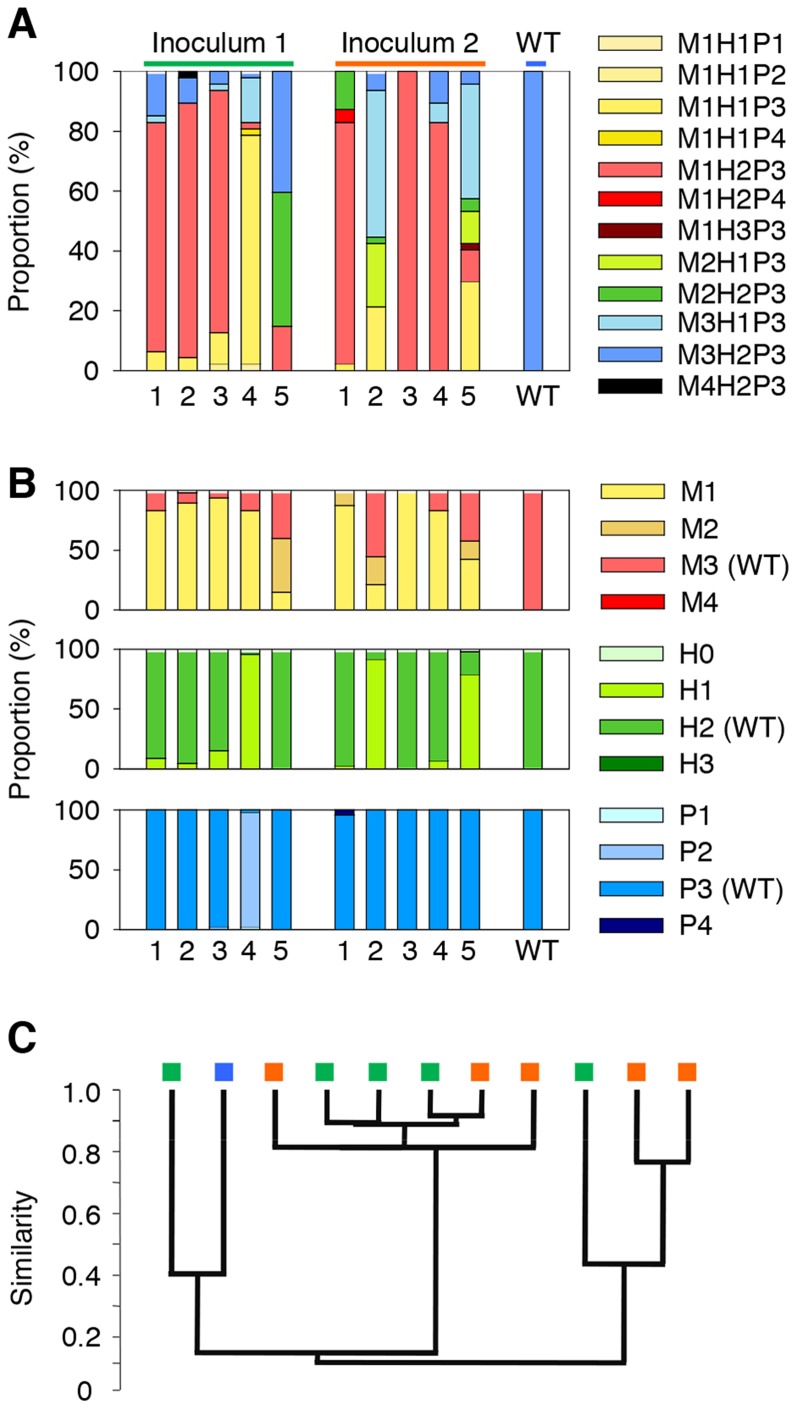
Phenotypic variant (PV) compositions of different sets of starvation populations. (A) The PV compositions of two sets of starvation populations, which started with the same inoculum, were analyzed for 33 days (n = 5). (B) Summaries of the phenotypic composition in terms of the motility (M), hemolysis (H), and proteolysis (P) activity levels are shown in. (C) Cluster analysis of the PV composition of each population. Green rectagulars, populations received inoculum 1; organge, populations received inoculum 2; blue, unstarved control. The analysis was based on the Bray-Curtis similarity and paired group methods.

PVs found in starved population may have been present in low numbers in the original population, or they may have been mutants derived from WT cells during starvation. Thus, we conducted five parallel starvation experiments using the same original population as the inoculum (Figure 3). We hypothesized that if these PVs were original members of the inoculum population and were selected during starvation, the compositions of these five surviving populations should be very similar.

The results showed that each population had a unique phenotypic composition after 33 days of starvation (Figure 3B), which suggested that these PVs were novel mutants that evolved independently in each population. However, similar trends were seen among the populations (Figure 3C). In both trials, mot-R PVs dominated the five populations, which indicates that a reduction in motility may provide more advantages than other mutations during early starvation. In some populations, hem-R PVs also increased in proportion (Figure 3B).

### Advantages of starvation survivors compared with wild-type cells

Certain PVs were observed repeatedly in the starvation experiments (Figures 2 and 3), which suggests that these PVs may have been more resistant to starvation-induced death, or that they may have been related to traits that made them more competitive than WT cells during starvation. To demonstrate that PVs performed better than the WT strain during starvation conditions, we selected three isolates, i.e., C30-2, D33-4, and B33-12, from three different survivor populations and performed competition tests with WT cells. C30-2 was isolated from the trial C 30-day population, and had reduced motility and enhanced proteolytic activity (M1H2P4 phenotype). D33-4 was isolated from the trial D 33-day population, and had reduced motility, reduced hemolysis, and increased proteolytic activity, and could not grow on TCBS plates (M1H1P4 phenotype). B33-12 was isolated from the trial B 33-day population, and had reduced motility and hemolysis activity levels (M1H1P3 phenotype).

**Figure 4:**
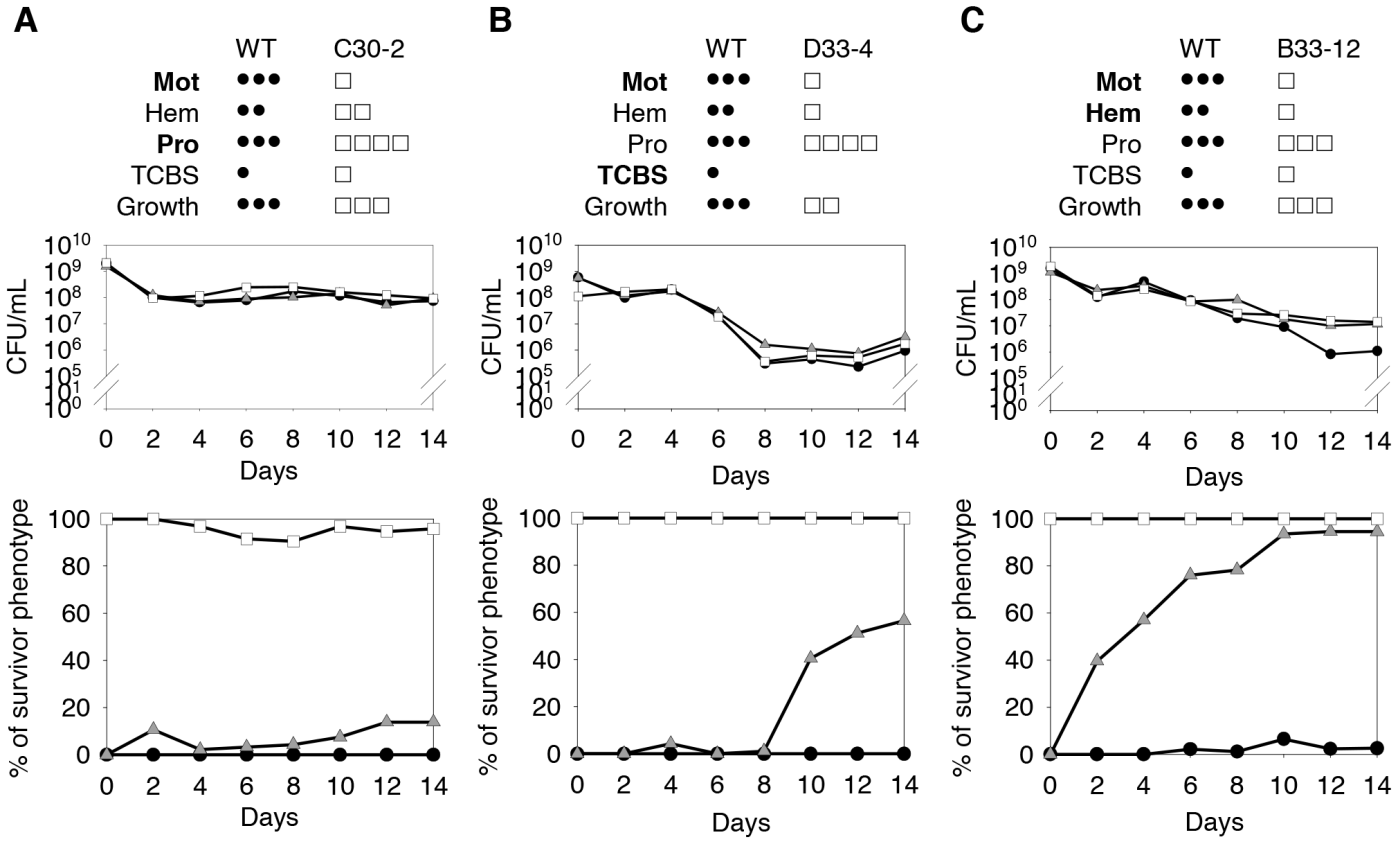
Competition between various phenotypic variants (PVs) derived from different survivor populations during starvation survival. Three PVs, i.e., C30-2 (A), D33-4 (B), and B33-12 (C), were competed with 93U204 cells in starvation conditions. The top panels show the population size and mutant phenotype dynamics. The bottom panels show the results of the competition experiments. For each mutant-wild-type (WT) pair, we measured the survival of the mutant-only (□), WT-only (•), or WT-mixed with 0.1% mutant (△) populations. The relative abundance of cells with the mutant phenotype was determined by examining 94 colonies in each trial. All three of the tested PVs increased their relative abundance after 14 days. Only one representative set of results (trial 1) is shown. The motility (Mot), hemolysis (Hem), proteolytic activity (Pro), growth on TCBS (TCBS), and growth on TSAS (growth) phenotype of each mutant was included in the figure and the characteristics used for differentiate mutant from WT colonies were shown in bold.

When cultured alone in PBS, the survival rates of all the tested PVs were similar to that of the progenitor control (Figure 4, upper), which indicated that they were not more resistant to starvation-induced death. When inoculated at 0.1% in a mixed culture with progenitor 93U204, the C30-2-like M1H2P4 cells exhibited a slow increase and reached 14% on day 14 (Figure 4A) (95% and 81% in the second and third trials), while D33-4-like M1H2P4 cells reached 56% on day 14 in mixed culture (Figure 4B) (79% and 62% in the second and third trials, respectively). B33-12-like M1H1P3 cells reached 40% on day 2 and continued to increase until they reached 95% on day 14 (Figure 4C) (49% and 79% in the second and third trials, respectively). The relative abundance of each mutant varied among trials, but they dominated from 0.1% in all three trails. In mutant monoculture, no isolates switched back to the WT phenotype. Similarly, only less than 6.5% of mutant-like isolates were detected in the 93U204 progenitor monoculture at the end of the experiments. Therefore, we conclude that all three tested PVs were more competitive than the WT cells at surviving starvation.

### Cell density, but not the number of cells, had a major effect on SID

**Figure 5:**
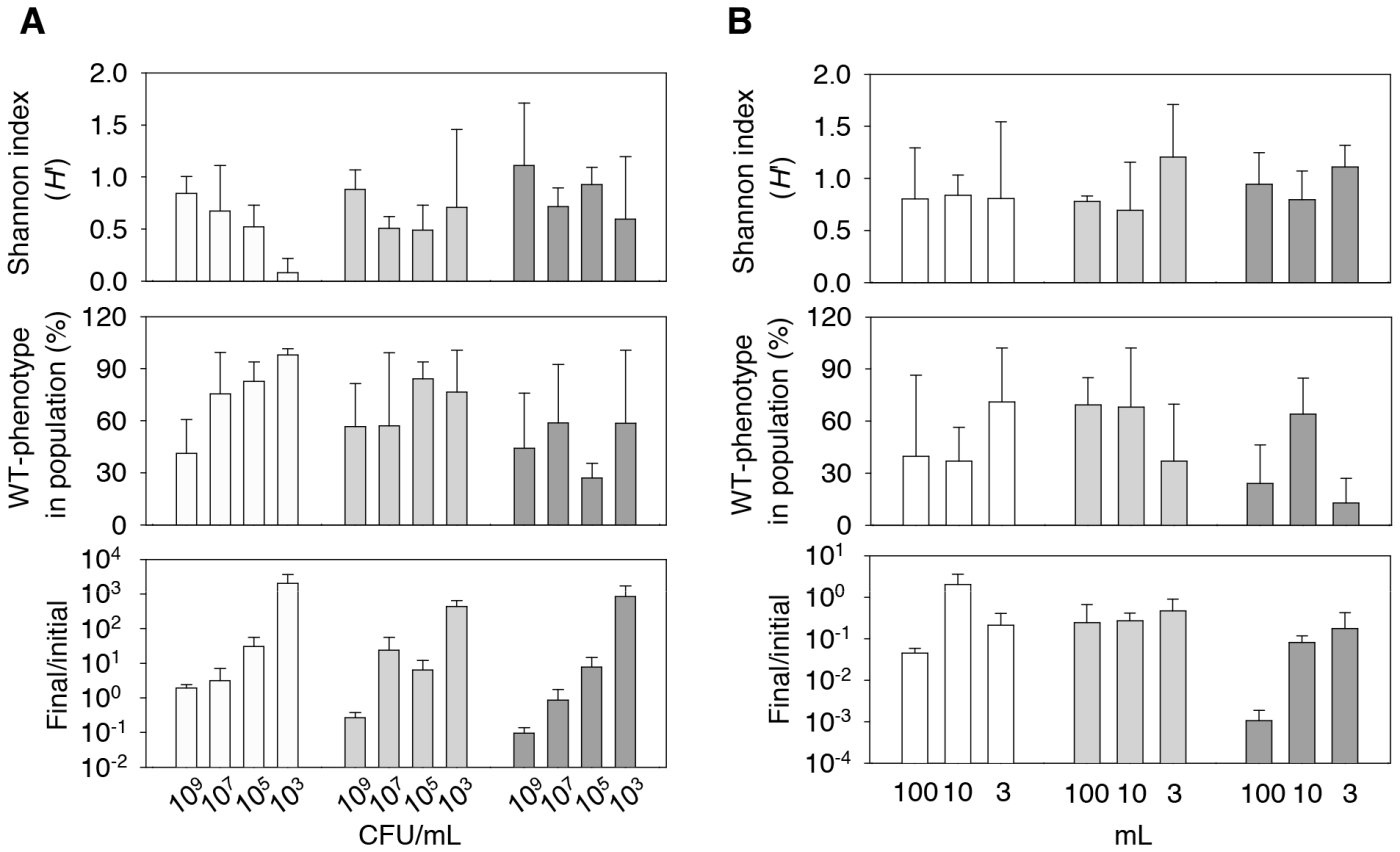
Effects of population density and population size on starvation-induced diversification. 93U204 cells were starved in 10 mL phosphate-buffered saline at densities of 10^9^, 10^7^, 10^5^, and 10^3^ CFU/mL (A), or in 100, 10 and 3 mL at a density of 10^9^ CFU/mL (B). The Shannon diversity index (top), percentage of cells with the wild-type (WT) phenotype (middle), and survival rate (bottom) of each population on days 14 (white bar), 28 (light grey bar), and 42 (dark grey bar) are shown. At day 14, phenotypic diversity (indicated by Shannon index) correlated positively with cell density, and percentage of WT and ratio of final to initial populations correlated negatively with cell density. After day 14, the trends in Shannon index and WT cell percentage were less apparent. Increased population size did not lead to more beneficial mutations in the population.

High density can be stressful for bacterial cells and lead to more mutations in the population. Thus, we tested the effect of cell density on the generation of SID. We examined SID at 10^9^, 10^7^, 10^5^, and 10^3^ CFU/mL. The PV diversity appeared to increase with density (Figure 5A). The Shannon index correlated positively with log-transformed cell density (linear regression, *r* = 0.76684, P = 0.0048). Both percentage of WT cells (*r*  = −0.81659, P = 0.0008) and ratio of final to initial population size (*r*  = −0.88628, P = 0.0001) correlated negatively with log-transformed cell density. However, this trend was only evident in the day 14 population and was absent on days 28 and 42, which suggests that the density effects were probably masked by saturation because of continuous diversification.

In these experiments, the increase in density was confounded by the increased absolute number of cells in the populations. Larger populations contained more cells so they were more likely to contain more mutants, which meant that there was a higher likelihood of beneficial mutations accumulating in these populations. In the next experiment, we fixed the starting cell density at 10^9^ CFU/mL and varied the volume of the medium among 100, 10, or 3 mL, before examining the effect of the population size on SID (Figure 5B). Neither Shannon index nor percentage of WT cell correlated significantly with medium volume. Therefore, we conclude that the cell density has a more important effect than the population size during phenotypic diversification in conditions of starvation.

### SID in seawater

**Figure 6:**
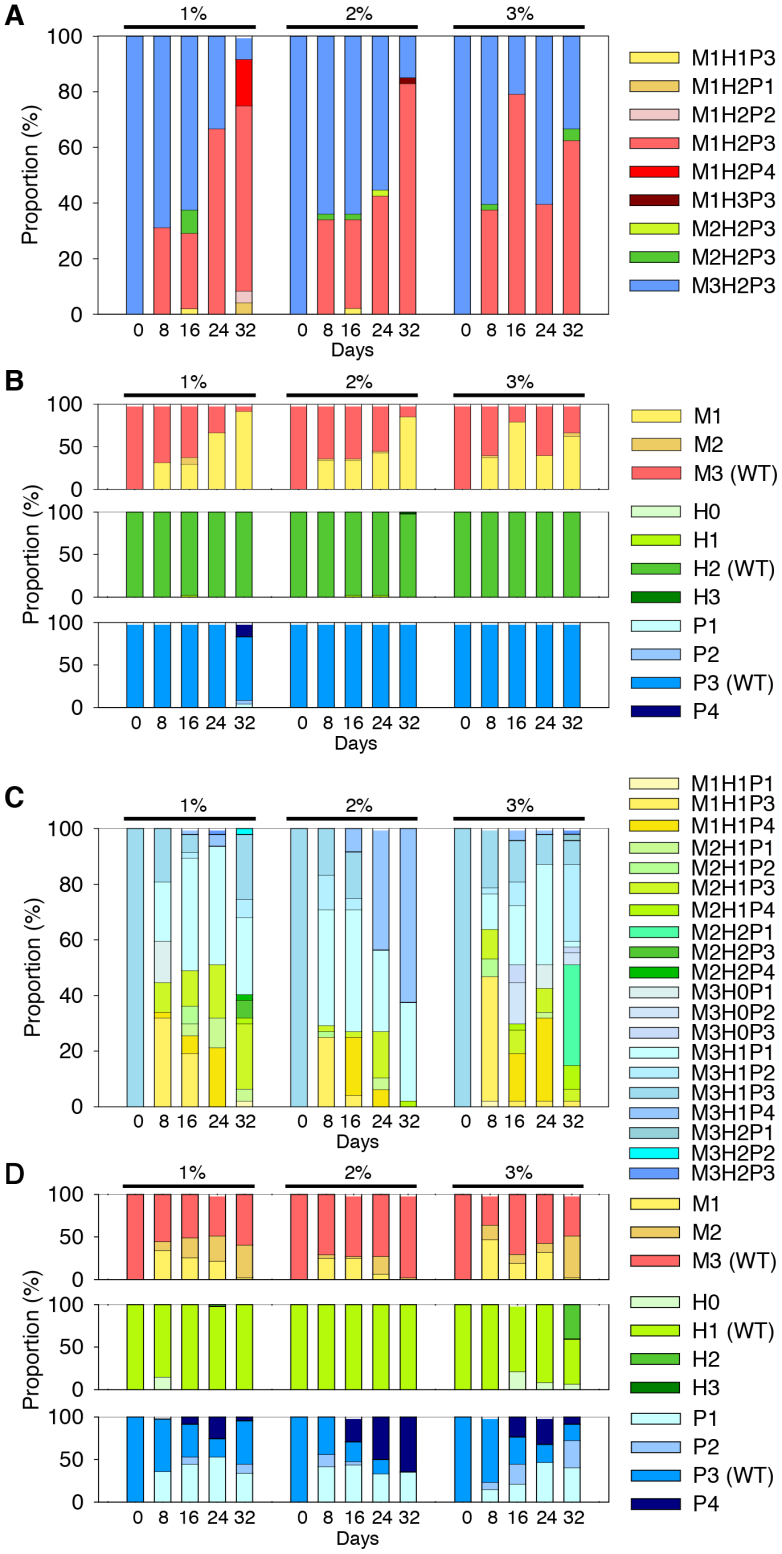
Starvation-induced diversification (SID) of *V. vulnificus* strains 93U204 and YJ016 in seawater. We tested the SID in natural seawater samples with salinities of 1%, 2%, or 3%. The results for 93U204 are shown in (A) and (B), while those for YJ016 are shown in (C) and (D). The starting concentration was 10^9^ CFU/mL. The phenotypic variant compositions (A and C) and the summarized population compositions (B and D) for motility (upper), hemolysis (middle), and proteolysis activity (lower) are also shown.

SID was readily observed in a laboratory setting, so we investigated whether this phenomenon also occurs in natural seawater. We starved *V. vulnificus* 93U204 cells in natural seawater samples, which were adjusted to salinities of 1%, 2%, or 3%, and we monitored any phenotypic changes (Figure 6A and B). The SID observed in natural seawater was comparable to that in PBS, which indicates that SID can occur in natural marine environments. For comparison, another *V. vulnificus* isolate YJ016 was also used in this starvation experiment (Figure 6C and D). SID was observed in both 93U204 and YJ016, although YJ016 diversified at a faster rate. The three populations cultured with different salinities had similar SID, which suggests that this phenomenon was not affected by salinity.

## Discussion

In this study, we determined the population dynamics of 93U204 during long-term starvation. The survival rates were similar to those reported in previous studies [14]–[16]. During the first week, there was a dramatic decrease in the viable population. Two weeks after inoculation, the population increased slightly, before the population entered a long and gradual decline (Figure 1). The life cycle of *E. coli* has been classified into lag, log, stationary, death, and long-term stationary phases [29]. *E. coli* cells grow rapidly during the log phase but the growth slows in the stationary phase when the nutrient supply decreases, and finally the cells progress to the death phase. Typically, >99% of the population lose their viability during the death phase, but the remaining cells can survive for several years with few declines in their cell numbers. The survival of *V. vulnificus* followed similar dynamics to those previously reported for *E. coli*, with an apparent death phase (rapid decrease) and a long-term stationary phase (slow decrease). A starved *E. coli* population maintained its size at approximately 10^6^ CFU/mL for over 5 years when sterile distilled water was supplied to maintain the volume and osmolarity [29]. In our experiments, *V. vulnificus* remained at approximately 10^6^ CFU/mL in PBS and 10^5^ CFU/mL in TSBS for 214 days (Figure 1B), while it remained at approximately 10^3^ CFU/mL in PBS for 1215 days. Therefore both species have comparable survival in longterm starvation.

After treating the stationary phase *E. coli* population with antibiotics to remove any dividing cells, some non-dividing cells were found to be dividing [30]. These cells were named “persisters” that allowed the population to survive in a harsh environment. Similar persisters may also play a role in the earlier stages during the survival of *V. vulnificus*, but they are not likely to be the major source of survivors because the “revived” persisters had the WT phenotype. The divergence between parallel populations and the fixed phenotype during passages strongly suggests that PVs are mutants derived from the progenitor cells. The cells acquired adaptive mutations during starvation, before subsequent selection, and they survived the stressful conditions.

Vibrios are known to enter the VBNC state under stress. In *V. vulnificus* the VBNC state could be induced through a temperature downshift to 5°C [31] or 4°C [32]. Although the cell viability seen in our starvation experiment may be similar to that in VBNC population, it is unlikely that the loss of viability is due to VBNC. Our experiments were conducted at 30°C, and no VBNC *V. vulnificus* has been reported at this temperature so far. An elevation of temperature to 21°C is sufficient to resuscitate VBNC *V. vulnificus* within 24 h [33]. We compared the property of 93U204 cells kept in PBS at either 30°C or at 4°C. After 9-day starvation treatment, most cells kept at 30°C co-stained with both propidium iodide and SYTO 9 (using LIVE/DEAD *Bac*Light Bacterial Viability Kit, for microscopy & quantitative assays, L7012, Life Technologies, Carlsbad, California, USA)(unpublished data), but co-staining was rarely seen in cells kept at 4°C. Addition of catalase is known to increase the survival of VBNC cells on agar plates [34]. Addition of catalase increased survival of cells kept at 4°C (with vs. without catalase, paired t-test, P = 0.038) by 4.6 ±3.2 times, but it had no effect on cells kept at 30°C (paired t-test, P = 0.4655). These data suggest that the loss of viability seen in our starvation experiments was not due to the induction of VBNC state.

Most cells that survived the death phase exhibited phenotypic changes (Figure 2). These changes were generally advantageous compared with the progenitor cells and they are referred to collectively as growth advantage in the stationary phase (GASP) phenotypes [29]. Cells that exhibit GASP phenotypes during starvation have been reported in bacteria such as *Campylobacter jejuni* [35], *Pseudomonas* [36], [37], *Geobacter* [38], and enterobacteria [39]. A GASP phenotype was also observed in *V. fischeri* [40]. A non-toxigenic *V. cholerae* isolate derived from a starved long-term slab culture was shown to have growth advantages compared with its toxigenic progenitors [41]. Mutations that lead to GASP have been studied in *E. coli* (reviewed in [29]). The loss of a functional *rpoS* may be advantageous in increasing amino acid utilization, while *lrp* and the *ybeJ*-*gltJKL* cluster encode the leucine-responsive protein and a high-affinity aspartate and glutamate transporter, respectively. In *Vibrio* spp., however, there is still no known genetic basis for GASP phenotypes.

Three types of strategies may facilitate bacterial survival during starvation: persister, scavenger, and replicator strategies. Cells that employ a persister strategy can persist during starvation conditions via physiological adaptation. These cells may have to trade their nutrient acquisition ability (nutritional competence) for self-preservation, according to the SPANC balance hypothesis [42]. Scavengers are cells that can increase their nutrient input, either by acquiring the ability to explore novel resources or by increasing their substrate-binding affinity or substrate-transporting ability. Replicators are cells that reduce their energy expenditure via mutation in functions that are not required for survival so they may have higher rates of replication. Scavengers and replicators may replicate faster than their progenitor cells, so they are more likely to dominate the surviving population.

Several systems have been used to study bacterial responses to nutrient limitation. Lenski’s laboratory have conducted a long-term evolution experiment (LTEE) with *E. coli* [43]. In this system, the bacteria are transferred to new broth each day. During the first few hours, replicators may have advantages, but the scavengers are favored over time. Persisters have an advantage only when the population experiences severe nutrient deprivation, which is not likely to happen in the LTEE. Therefore, replicators and scavengers are expected to dominate in this system. The mean fitness increased by approximately 35% in the 2000-generation population [44]. The adaptation led to a decrease in the lag time before growth and an increase in the maximum growth rate, although the death rate and the nutrient concentration required to support half the maximum rate did not change significantly. Therefore, replicators were favored in this system.

Ferenci’s laboratory pioneered the use of a chemostat system to study the response and evolution of *E. coli* in a constant low-nutrient environment [45]. The nutrient concentrations were maintained at stable low levels, so the scavengers had an advantage over replicators and persisters. In this system, an *rpoS* mutant rapidly became dominant within the first 3 days [46]. Growth in these conditions is highly *rpoD*-dependent, which supports *rpoS* mutations [47]. Mutations in the *rpoS*, *mlc*, *malT,* and *mgl* have been demonstrated to increase the expression of *ompF*, *lamB*, *mglBAC,* and *ptsG*, which improves high-affinity glucose uptake [48]–[50]. Thus, the scavengers dominated this system.

In our study, we starved the cells in PBS from the start, so persisters may have possessed advantages compared with other cells during the first few days. Dead cells accumulated rapidly over time and the surviving cells actively utilized the nutrients released from the dead cells, so they gradually increased in number and dominated the population. The first types of PVs that appeared in the starved populations were mot-R PVs (Figures 2 and 6). Reductions or loss of motility after starvation have been reported in diverse bacteria, including *Rhizobium meliloti* [51], *V. angustum* [52], and unidentified marine bacteria [53]. Flagellar motility requires much energy, so a reduction in bacterial motility has the benefit of saving energy, which is consistent with the recently proposed Black Queen hypotheses [54]. However, these PVs did not have an increased growth rate in TSBS. It is likely that these PVs lacked a growth advantage, or they may only possess a growth advantage in low-nutrient conditions. During a stringent response, *E. coli* downregulates the expression of flagellar genes [55], [56]. Recently, it was reported that *V. vulnificus* downregulates its flagellar regulator FlhF in the stationary phase via the quorum sensing master regulator SmcR [57]. This indicates that cells are not motile in the stationary phase so those that lose motility will not be selected against and will tend to be detected. Without appropriate evidence, we were not able to tell whether the rapid appearance of mot-R PVs was a result of a replicator or scavenger strategy. However, based on the presence of large numbers of dead cells, a scavenger strategy was more favored.

In the present study, the average proteolytic activity of the 93U204 survivor populations remained unchanged or increased, but the hemolytic activity decreased with time (Figure 2). These trends may represent a trade-off between using energy to produce the corresponding proteins and acquiring nutrients from dead cells with these proteins. In the set of experiment shown in Figure 6B, the proteolytic activity and hemolytic activity of the survivors remained largely unchanged, although mot-R PVs still dominated. We hypothesize that a reduction in motility is a more reliable strategy for increasing fitness, whereas the advantage of manipulating exoenzyme production is dependent on the environment and the competing bacteria. Similarly, in YJ016, PVs with reduced or enhanced proteolytic activity increased their relative abundance (Figure 6D), so other factors may be more important when surviving starvation.

In the competition experiments, all three PVs dominated the progenitor cells in all three trials. However, there were high variations in their proportions in the final population. The mutants had advantages over the progenitor cells, but newly emerged mutations in the populations could potentially affect the results of the competition, although these new mutations were practically undetectable because we only examined a limited number of phenotypic features. This observation emphasizes the dynamic nature of evolution under starvation conditions, where the exact composition of the starved population will be changing constantly.

In conclusion, the dynamics of *V. vulnificus* population under conditions of starvation were very similar to those of *E. coli,* which may be common in Gammaproteobacteria. In addition, SID was observed in populations cultured in PBS, which also occurred with a wide range of salinities. We suggest that SID is likely to occur in natural settings, which may be the mechanism that maintains the high phenotypic diversity observed in natural populations. Further investigations are required to determine whether this mechanism is linked to the emergence of pathogenic strains from environmental bacteria.

## References

[1] Jones MK, Oliver JD (2009) *Vibrio vulnificus*: disease and pathogenesis. Infect Immun 77: 1723–1733 10.1128/IAI.01046-08 19255188 PMC2681776

[2] Tison DL, Nishibuchi M, Greenwood JD, Seidler RJ (1982) *Vibrio vulnificus* biogroup 2: new biogroup pathogenic for eels. Appl Environ Microbiol 44: 640–646. 7138004 10.1128/aem.44.3.640-646.1982 PMC242070

[3] Mahmud ZH, Wright AC, Mandal SC, Dai J, Jones MK, et al (2010) Genetic characterization of *Vibrio vulnificus* strains from tilapia aquaculture in Bangladesh. Appl Environ Microbiol 76: 4890–4895 10.1128/AEM.00636-10 20495047 PMC2901738

[4] Dalsgaard I, Høi L, Siebeling RJ, Dalsgaard A (1999) Indole-positive *Vibrio vulnificus* isolated from disease outbreaks on a Danish eel farm. Dis Aquat Org 35: 187–194 10.3354/dao035187 10228875

[5] Fouz B, Larsen JL, Amaro C (2006) *Vibrio vulnificus* serovar A: an emerging pathogen in European anguilliculture. J Fish Dis 29: 285–291 10.1111/j.1365-2761.2006.00719.x 16677318

[6] Daniels NA (2011) *Vibrio vulnificus* oysters: pearls and perils. Clin Infect Dis 52: 788–792 10.1093/cid/ciq251 21367733

[7] Gopal S, Otta SK, Kumar S, Karunasagar I, Nishibuchi M, et al (2005) The occurrence of *Vibrio* species in tropical shrimp culture environments; implications for food safety. Int J Food Microbiol 102: 151–159 10.1016/j.ijfoodmicro.2004.12.011 15992615

[8] Arias CR, Pujalte MJ, Garay E, Aznar R (1998) Genetic relatedness among environmental, clinical, and diseased-eel *Vibrio vulnificus* isolates from different geographic regions by ribotyping and randomly amplified polymorphic DNA PCR. Appl Environ Microbiol 64: 3403–3410. 9726889 10.1128/aem.64.9.3403-3410.1998 PMC106739

[9] Wong HC, Chang CN (2005) Hydrophobicity, cell adherence, cytotoxicity, and enterotoxigenicity of starved *Vibrio parahaemolyticus* . J Food Prot 68: 154–156. 15690818 10.4315/0362-028x-68.1.154

[10] Hengge R (2011) The general stress response in Gram-negative bacteria. In: Storz G, Hengge R, editors. Bacterial Stress Responses. ASM Press. pp. 251–290.

[11] Foster PL (2007) Stress-induced mutagenesis in bacteria. Crit Rev Biochem Mol Biol 42: 373–397 10.1080/10409230701648494 17917873 PMC2747772

[12] Wright BE (2004) Stress-directed adaptive mutations and evolution. Mol Microbiol 52: 643–650 10.1111/j.1365-2958.2004.04012.x 15101972

[13] Gonzalez C, Hadany L, Ponder RG, Price M, Hastings PJ, et al (2008) Mutability and importance of a hypermutable cell subpopulation that produces stress-induced mutants in *Escherichia coli* . PLoS Genet 4: e1000208 10.1371/journal.pgen.1000208 18833303 PMC2543114

[14] Biosca EG, Amaro C, Marco-Noales E, Oliver JD (1996) Effect of low temperature on starvation-survival of the eel pathogen *Vibrio vulnificus* biotype 2. Appl Environ Microbiol 62: 450–455. 8593047 10.1128/aem.62.2.450-455.1996 PMC167812

[15] Marco-Noales E, Biosca EG, Amaro C (1999) Effects of salinity and temperature on long-term survival of the eel pathogen *Vibrio vulnificus* biotype 2 (serovar E). Appl Environ Microbiol 65: 1117–1126. 10049871 10.1128/aem.65.3.1117-1126.1999 PMC91152

[16] Hülsmann A, Rosche TM, Kong IS, Hassan HM, Beam DM, et al (2003) RpoS-dependent stress response and exoenzyme production in *Vibrio vulnificus* . Appl Environ Microbiol 69: 6114–6120. 14532069 10.1128/AEM.69.10.6114-6120.2003 PMC201245

[17] Amel BKN, Amine B, Amina B (2008) Survival of *Vibrio fluvialis* in seawater under starvation conditions. Microbiol Res 163: 323–328 10.1016/j.micres.2006.06.006 16870413

[18] González-Escalona N, Fey A, Höfle MG, Espejo RT, A Guzmán C (2006) Quantitative reverse transcription polymerase chain reaction analysis of *Vibrio cholerae* cells entering the viable but non-culturable state and starvation in response to cold shock. Environ Microbiol 8: 658–666 10.1111/j.1462-2920.2005.00943.x 16584477

[19] Chen SY, Jane WN, Chen YS, Wong HC (2009) Morphological changes of *Vibrio parahaemolyticus* under cold and starvation stresses. Int J Food Microbiol 129: 157–165 10.1016/j.ijfoodmicro.2008.11.009 19101053

[20] Ostling J, Flärdh K, Kjelleberg S (1995) Isolation of a carbon starvation regulatory mutant in a marine *Vibrio* strain. J Bacteriol 177: 6978–6982. 7592494 10.1128/jb.177.23.6978-6982.1995 PMC177569

[21] Vattakaven T, Bond P, Bradley G, Munn CB (2006) Differential effects of temperature and starvation on induction of the viable-but-nonculturable state in the coral pathogens *Vibrio shiloi* and *Vibrio tasmaniensis* . Appl Environ Microbiol 72: 6508–6513 10.1128/AEM.00798-06 17021199 PMC1610280

[22] Chen CY, Chao CB, Bowser PR (2006) Infection of tilapia *Oreochromis* sp. by *Vibrio vulnificus* in freshwater and low-salinity environments. Journal of the World Aquaculture Society 37: 82–88 10.1111/j.1749-7345.2006.00010.x

[23] Chen CY, Wu KM, Chang YC, Chang CH, Tsai HC, et al (2003) Comparative genome analysis of *Vibrio vulnificus*, a marine pathogen. Genome Res 13: 2577–2587 10.1101/gr.1295503 14656965 PMC403799

[24] Herigstad B, Hamilton M, Heersink J (2001) How to optimize the drop plate method for enumerating bacteria. J Microbiol Methods 44: 121–129. 11165341 10.1016/s0167-7012(00)00241-4

[25] Park KJ, Kang MJ, Kim SH, Lee HJ, Lim JK, et al (2004) Isolation and characterization of *rpoS* from a pathogenic bacterium, *Vibrio vulnificus*: role of sigmaS in survival of exponential-phase cells under oxidative stress. J Bacteriol 186: 3304–3312 10.1128/JB.186.11.3304-3312.2004 15150215 PMC415748

[26] Hill WE, Keasler SP, Trucksess MW, Feng P, Kaysner CA, et al (1991) Polymerase chain reaction identification of *Vibrio vulnificus* in artificially contaminated oysters. Appl Environ Microbiol 57: 707–711. 2039231 10.1128/aem.57.3.707-711.1991 PMC182783

[27] Chen C (2005) Regulatory roles of the sigma factors RpoS and RpoN in the pathogenesis of *Vibrio vulnificus* YJ016. [master thesis]. Taiwan: Chung-Shan Medical University.

[28] Hammer Ø, Harper DAT, Ryan PD (2001) PAST: Paleontological statistics software: package for education and data Analysis. Palaeontologia Electronica: 1–9.

[29] Finkel SE (2006) Long-term survival during stationary phase: evolution and the GASP phenotype. Nat Rev Microbiol 4: 113–120 10.1038/nrmicro1340 16415927

[30] Roostalu J, Jõers A, Luidalepp H, Kaldalu N, Tenson T (2008) Cell division in *Escherichia coli* cultures monitored at single cell resolution. BMC Microbiology 8: 68 10.1186/1471-2180-8-68 18430255 PMC2377270

[31] Oliver JD, Bockian R (1995) In vivo resuscitation, and virulence towards mice, of viable but nonculturable cells of *Vibrio vulnificus* . Appl Environ Microbiol 61: 2620–2623. 7618873 10.1128/aem.61.7.2620-2623.1995 PMC167533

[32] Smith B, Oliver JD (2006) In situ and in vitro gene expression by *Vibrio vulnificus* during entry into, persistence within, and resuscitation from the viable but nonculturable state. Appl Environ Microbiol 72: 1445–1451 10.1128/AEM.72.2.1445-1451.2006 16461698 PMC1392903

[33] Oliver JD, Hite F, McDougald D, Andon NL, Simpson LM (1995) Entry into, and resuscitation from, the viable but nonculturable state by *Vibrio vulnificus* in an estuarine environment. Appl Environ Microbiol 61: 2624–2630. 7618874 10.1128/aem.61.7.2624-2630.1995 PMC167534

[34] Kong IS, Bates TC, Hülsmann A, Hassan H, Smith BE, et al (2004) Role of catalase and *oxyR* in the viable but nonculturable state of *Vibrio vulnificus* . FEMS Microbiol Ecol 50: 133–142 10.1016/j.femsec.2004.06.004 19712354

[35] Martínez-Rodriguez A, Kelly AF, Park SF, Mackey BM (2004) Emergence of variants with altered survival properties in stationary phase cultures of *Campylobacter jejuni* . Int J Food Microbiol 90: 321–329. 14751687 10.1016/s0168-1605(03)00325-8

[36] Silby MW, Giddens SR, Mahanty HK (2005) Mutation of a LysR-type regulator of antifungal activity results in a growth advantage in stationary phase phenotype in *Pseudomonas aureofaciens* PA147-2. Appl Environ Microbiol 71: 569–573 10.1128/AEM.71.1.569-573.2005 15640239 PMC544236

[37] Tark M, Tover A, Tarassova K, Tegova R, Kivi G, et al (2005) A DNA polymerase V homologue encoded by TOL plasmid pWW0 confers evolutionary fitness on *Pseudomonas putida* under conditions of environmental stress. J Bacteriol 187: 5203–5213 10.1128/JB.187.15.5203-5213.2005 16030214 PMC1196032

[38] Helmus RA, Liermann LJ, Brantley SL, Tien M (2012) Growth advantage in stationary-phase (GASP) phenotype in long-term survival strains of *Geobacter sulfurreducens* . FEMS Microbiol Ecol 79: 218–228 10.1111/j.1574-6941.2011.01211.x 22029575

[39] Bacun-Druzina V, Cagalj Z, Gjuracic K (2007) The growth advantage in stationary-phase (GASP) phenomenon in mixed cultures of enterobacteria. FEMS Microbiol Lett 266: 119–127 10.1111/j.1574-6968.2006.00515.x 17233722

[40] Petrun B, Lostroh CP (2013) *Vibrio fischeri* exhibit the growth advantage in stationary-phase phenotype. Can J Microbiol 59: 130–135 10.1139/cjm-2012-0439 23461521

[41] Paul K, Ghosh A, Sengupta N, Chowdhury R (2004) Competitive growth advantage of nontoxigenic mutants in the stationary phase in archival cultures of pathogenic *Vibrio cholerae* strains. Infect Immun 72: 5478–5482 10.1128/IAI.72.9.5478-5482.2004 15322049 PMC517435

[42] Ferenci T, Spira B (2007) Variation in stress responses within a bacterial species and the indirect costs of stress resistance. Annals of the New York Academy of Sciences 1113: 105–113 10.1196/annals.1391.003 17483210

[43] Lenski RE, Travisano M (1994) Dynamics of adaptation and diversification: a 10,000-generation experiment with bacterial populations. Proc Natl Acad Sci USA 91: 6808–6814. 8041701 10.1073/pnas.91.15.6808 PMC44287

[44] Vasi F, Travisano M, Lenski RE (1994) Long-term experimental evolution in *Escherichia coli*. II: Changes in life-history traits during adaptative to a seasonal environment. The American naturalist 144: 432–456.

[45] Death A, Notley L, Ferenci T (1993) Derepression of LamB protein facilitates outer membrane permeation of carbohydrates into *Escherichia coli* under conditions of nutrient stress. J Bacteriol 175: 1475–1483. 8444809 10.1128/jb.175.5.1475-1483.1993 PMC193235

[46] Notley-McRobb L, Seeto S, Ferenci T (2003) The influence of cellular physiology on the initiation of mutational pathways in *Escherichia coli* populations. Proc Biol Sci 270: 843–848 10.1098/rspb.2002.2295 12737663 PMC1691312

[47] Ferenci T (2003) What is driving the acquisition of *mutS* and *rpoS* polymorphisms in *Escherichia coli*? Trends Microbiol 11: 457–461. 14557028 10.1016/j.tim.2003.08.003

[48] Notley-McRobb L, Ferenci T (1999) Adaptive *mgl*-regulatory mutations and genetic diversity evolving in glucose-limited *Escherichia coli* populations. Environ Microbiol 1: 33–43. 11207716 10.1046/j.1462-2920.1999.00002.x

[49] Notley-McRobb L, Ferenci T (1999) The generation of multiple co-existing *mal*-regulatory mutations through polygenic evolution in glucose-limited populations of *Escherichia coli* . Environ Microbiol 1: 45–52. 11207717 10.1046/j.1462-2920.1999.00003.x

[50] Notley-McRobb L, Ferenci T (2000) Experimental analysis of molecular events during mutational periodic selections in bacterial evolution. Genetics 156: 1493–1501. 11102352 10.1093/genetics/156.4.1493 PMC1461358

[51] Wei X, Bauer WD (1998) Starvation-induced changes in motility, chemotaxis, and flagellation of *Rhizobium meliloti* . Appl Environ Microbiol 64: 1708–1714. 9572940 10.1128/aem.64.5.1708-1714.1998 PMC106219

[52] Stretton S, Danon SJ, Kjelleberg S, Goodman AE (1997) Changes in cell morphology and motility in the marine *Vibrio* sp. strain S14 during conditions of starvation and recovery. FEMS Microbiology Letters 146: 23–29 10.1016/S0378-1097(96)00387-4

[53] Amy PS, Morita RY (1983) Starvation-survival patterns of sixteen freshly isolated open-ocean bacteria. Appl Environ Microbiol 45: 1109–1115. 16346231 10.1128/aem.45.3.1109-1115.1983 PMC242413

[54] Morris JJ, Lenski RE, Zinser ER (2012) The Black Queen Hypothesis: evolution of dependencies through adaptive gene loss. MBio 3 10.1128/mBio.00036-12 PMC3315703 22448042

[55] Durfee T, Hansen A-M, Zhi H, Blattner FR, Jin DJ (2008) Transcription profiling of the stringent response in *Escherichia coli* . J Bacteriol 190: 1084–1096 10.1128/JB.01092-07 18039766 PMC2223561

[56] Lemke JJ, Durfee T, Gourse RL (2009) DksA and ppGpp directly regulate transcription of the *Escherichia coli* flagellar cascade. Mol Microbiol 74: 1368–1379 10.1111/j.1365-2958.2009.06939.x 19889089 PMC2806482

[57] Kim SM, Lee DH, Choi SH (2012) Evidence that the *Vibrio vulnificus* flagellar regulator FlhF is regulated by a quorum sensing master regulator SmcR. Microbiology (Reading, Engl) 158: 2017–2025 10.1099/mic.0.059071-0 22679105

